# Stefan Borowiecki (1881–1937)

**DOI:** 10.1007/s00415-019-09334-9

**Published:** 2019-04-29

**Authors:** Krystyna Makowska

**Affiliations:** Department of Clinical Physiology, Faculty of Veterinary Medicine, University of Warmia and Mazury in Olsztyn, Oczapowskiego Str 13, 10-718 Olsztyn, Poland

## Introduction

**Fig. 1:**
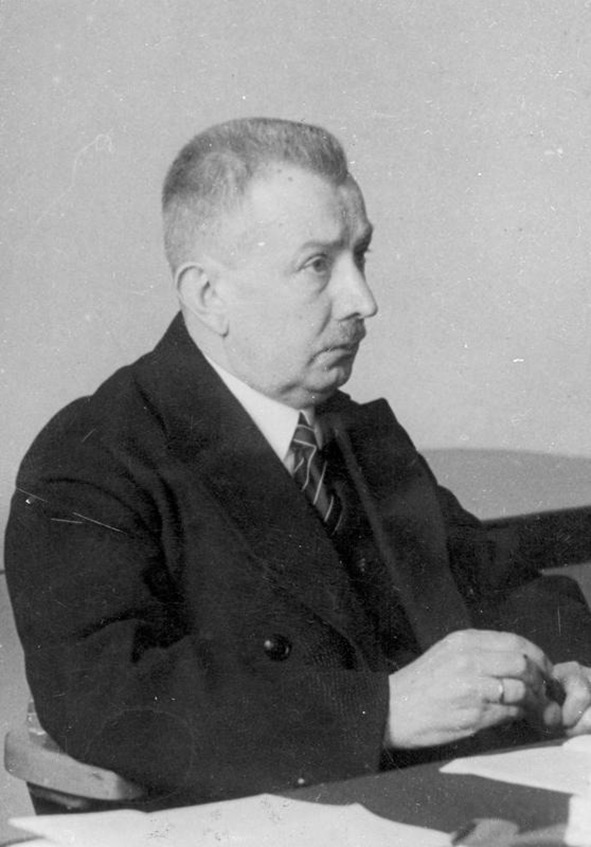
Stefan Borowiecki. Photo from public domain

Stefan Borowiecki (Fig. 1) was born on August 20, 1881 in Warsaw in an intellectual family. His father was a state councilor and customs official [1]. In his hometown, young Stefan attended elementary and philological middle school. In 1900, he started to study at the Medical Faculty of Warsaw University [2]. He finished his studies in 1905, but did not pass the state medical examination because of the Polish Revolution of 1905, student strikes and the closure of the university [3].

Following university, Borowiecki took a temporary job in the internal diseases department at the Baby Jesus Clinical Hospital in Warsaw, and soon afterwards at a psychiatric hospital in Kochanowka near Lodz [2, 4]. In Kochanowka, Borowiecki wrote his first scientific work on the subject of dissociated sensory loss and psychological disturbances during syphilis. He also observed mechanisms of psychological trauma caused by the events connected with fighting during the 1905 Revolution. These observations were some of the first studies on what now might be termed posttraumatic stress disorder.

In the meantime (1906), he went to Kazan, where he passed the medical state examination [4]. A year later, he went to Cracow, where he came in contact with the famous neurologist Jan Piltz [5]. With his help, Borowiecki pursued foreign scientific internships. Initially, he worked in Switzerland, with noted neurologists and psychiatrists including Friedrich Ris, Constantioc von Monakov [6], Paul Bleuer and Carl Jung [2, 4].

Under the leadership of Monakov, Borowiecki carried out research studies on the anatomy, cytoarchitecture and pathology of neural pathways and tracts within pons Varolii. He described for the first time the groups of neurons in the pons, which did not show the direct connections with either the cortex or cerebellum, identified new neuronal tracts within the pons, and studied on transneuronal degeneration of pontine neurons after an experimental damage [7]. It should be pointed out that in the first years of the twentieth century the pons Varolli was considered to be an intermediate station in the conduction of motor stimuli. Borowiecki’s studies shed new light on the functions of this part of the nervous system [7].

On completion of the studies on the pons Varolli in Switzerland, Borowiecki went to Paris, where he worked at neurological departments headed by distinguished neurologists such as Fulgence Raymond, Joseph Dejerine and Joseph Babinski [2]. In 1910, Borowiecki returned to Cracow and took a job at the Clinic of Neuronal and Mental Disease of Jagiellonian University headed by Jan Piltz. In 1913, he went for a half-year internship to the neurological and psychiatric Clinic in University of Berlin [4].

Two main areas of scientific interests can be distinguished in Borowiecki’s work. One was neurology [7]. Borowiecki was one of the pioneers of studies of the mechanisms and signs of gunshot injuries of peripheral nerve fibers and their treatment during World War I. He published an article entitled “Some insights in the course of gunshot disorders of the peripheral nerves” in 1915 [8]. Because thousands of soldiers were wounded during the war, Borowiecki’s studies had great practical significance. At that time, Borowiecki also studied birth defects of the central nervous system. Among others, he described the macroscopic and microscopic changes in arhinencephaly and carried out a classification of birth defects within the cerebrum depending on anatomo-pathological changes [7]. Thanks to these studies, Borowiecki obtained the habilitation.

His second area of scientific interests was psychiatry [9]. During the internship in Switzerland, Borowiecki had come in contact with the theory of psychoanalysis developed by Sigmund Freud and he became a strong supporter of it. In 1912, during the Second Convention of Polish Neurologists, Psychiatrists and Psychologists, Borowiecki gave a lecture in which he argued that psychoanalysis was the only way allowing so profound a penetration of the psyche in health and disease [9]. Borowiecki also studied posttraumatic stress disorder in soldiers of World War I, mechanisms of persecutory delusions and the roles of inheritance in mental diseases [2].

During the World War I, Borowiecki was a military doctor in the army of the Austro-Hungarian Empire, and in 1919, after Poland regained its independence, he was appointed chief of the department of nerve and mental diseases at Saint Lazarus Hospital in Cracow. In 1920, he served as a major surgeon during the Polish–Soviet War [1]. After the demobilization, Borowiecki was appointed the head of the Department of Neurology and Psychiatry at University in Poznan, which was at the time in the early stages of organization. Borowiecki organized the neurological clinic, as well as biochemical and neuropathological laboratories [1, 2], and also conducted lectures for students on neurology and psychiatry. He received the title of full professor in 1923 and was the dean of faculty of Medicine in 1929–1930. He was active in numerous scientific societies and scientific journals. He was the president of Polish Neurological Societies and of the Medical Society of Friends of Learning in Poznan [1, 2, 4]. Simultaneously, Borowiecki carried out intensive research in neurology and psychiatry. He studied mechanisms of Alzheimer’s disease, paresis caused by tumors in the central nervous system, and changes in the brain after skull injuries [4]. He continued studies concerning the inheritance of mental diseases and of posttraumatic stress disorder [7].

His intensive work contributed to the deterioration of his health. He died suddenly of heart attack on September 8, 1937 in Poznan [3]. The attitude of Borowiecki toward his patients and work is reflected most clearly in the words of his friend, the psychiatrist Witold Łuniewski, who said: “Appreciation, respect and sympathy, which he reached in people were acquired through the value of his character and his sincere fondness for truth and goodness that he so faithfully served all his life” [4].
